# When Droplets Can “Think”: Intelligent Testing in Digital Microfluidic Chips

**DOI:** 10.3390/bios16010003

**Published:** 2025-12-19

**Authors:** Zhijie Luo, Shaoxin Li, Wufa Long, Rui Chen, Jianhua Zheng

**Affiliations:** College of Artificial Intelligence, Zhongkai University of Agriculture and Engineering, Guangzhou 510225, China; luozhijie@zhku.edu.cn (Z.L.); lishaoxin@zhku.edu.cn (S.L.); longwufa@zhku.edu.cn (W.L.); chenrui01@zhku.edu.cn (R.C.)

**Keywords:** digital microfluidic biochips, online testing, path planning, priority strategy, improved sparrow search algorithm

## Abstract

Digital microfluidic biochips (DMFBs) find extensive applications in biochemical experiments, medical diagnostics, and safety-critical domains, with their reliability dependent on efficient online testing technologies. However, traditional random search algorithms suffer from slow convergence and susceptibility to local optima under complex fluidic constraints. This paper proposes a hybrid optimization method based on priority strategy and an improved sparrow search algorithm for DMFB online test path planning. At the algorithmic level, the improved sparrow search algorithm incorporates three main components: tent chaotic mapping for population initialization, cosine adaptive weights together with Elite Opposition-based Learning (EOBL) to balance global exploration and local exploitation, and a Gaussian perturbation mechanism for fine-grained refinement of promising solutions. Concurrently, this paper proposes an intelligent rescue strategy that integrates global graph-theoretic pathfinding, local greedy heuristics, and space–time constraint verification to establish a closed-loop decision-making system. The experimental results show that the proposed algorithm is efficient. On the standard 7 × 7–15 × 15 DMFB benchmark chips, the shortest offline test path length obtained by the algorithm is equal to the length of the Euler path, indicating that, for these regular layouts, the shortest test path has reached the known optimal value. In both offline and online testing, the shortest paths found by the proposed method are better than or equal to those of existing mainstream algorithms. In particular, for the 15 × 15 chip under online testing, the proposed method reduces the path length from 543 and 471 to 446 compared with the IPSO and IACA algorithms, respectively, and reduces the standard deviation by 53.14% and 39.4% compared with IGWO in offline and online testing.

## 1. Introduction

Digital Microfluidic Biochips (DMFBs) [1], also known as Lab on a Chip, are programmable microfluidic platforms that manipulate discrete droplets through the Electrowetting-on-Dielectric (EWOD) [2,3] mechanism, achieving high-precision control of droplet transportation and reaction processes. Compared with conventional biochemical analysis systems, DMFBs offer several remarkable advantages, including a high level of integration, low sample consumption, rapid reaction speed, and high automation [4]. These features have enabled their widespread application in fields such as biomedical detection [5], DNA analysis [6], and drug screening [7]. However, as the chip scale and experimental complexity continue to increase, the demand for operational reliability and stability has become considerably more stringent. Any malfunction in droplet manipulation or electrode control may cause experiment interruption or even complete task failure. Therefore, ensuring the safety and reliability of DMFBs under dynamic operating conditions has become a critical research challenge.

Traditional testing of DMFBs primarily relies on offline testing methods, which are typically based on graph-theoretic path planning models [8,9]. The core idea is to employ a test droplet to traverse the electrode array to detect potential faults. However, such methods can only identify static defects that occur during the fabrication stage and are ineffective in addressing dynamic faults that arise during runtime. To overcome this limitation, [10] proposed that the test path should not only cover all electrodes but also traverse all inter-electrode boundaries, and for the first time introduced the Euler path model into DMFBs test path planning. This approach effectively improved the fault coverage rate. Nevertheless, the Euler path-based method requires relatively long waiting times under dynamic conditions, making it difficult to meet the real-time requirements of online testing.

As research has deepened, scholars have begun to introduce intelligent optimization algorithms to enhance the efficiency and robustness of test path planning. Early studies primarily employed the Ant Colony Optimization (ACO) algorithm [11,12] to optimize test paths, which performs global search by simulating the pheromone communication mechanism of ants, thereby improving path optimization performance to a certain extent. However, since the test path planning problem is essentially NP-hard [8,13], ACO still suffers from high randomness, susceptibility to local optima, and slow convergence in high-dimensional complex spaces. To overcome these limitations, subsequent studies introduced the Particle Swarm Optimization (PSO) algorithm [14,15] and the genetic algorithm (GA) [16,17]. PSO improves search speed through a population-based collaboration mechanism but tends to exhibit premature convergence in dynamic constraint environments. Although GA possesses strong global optimization capability, its crossover and mutation operators are highly sensitive to parameter settings, leading to unstable outcomes. Moreover, as the chip scale increases or fluidic constraints dynamically change, the performance of these algorithms deteriorates significantly, making it difficult to satisfy the requirements for real-time performance and robustness in online testing of DMFB systems.

On the other hand, to further enhance testing efficiency, several studies have proposed multi-droplet parallel testing strategies [18,19,20]. These approaches perform parallel path scanning by simultaneously injecting multiple test droplets onto the chip, thereby significantly reducing overall testing time. In addition, some studies have introduced pipeline scanning [21] and built-in self-test (BIST) architectures [22] to improve the efficiency of offline testing. However, parallel testing requires complex synchronization and scheduling mechanisms, and when conflicts or resource contention occur among test paths, it can easily result in droplet blockage and deadlock issues. Furthermore, built-in self-test methods require the inclusion of logic gates and control circuits, which increase system design complexity and power consumption. These drawbacks make such approaches unsuitable for direct application in dynamic online testing environments.

In summary, although existing studies have made notable progress in path planning and parallel testing, there remains a lack of comprehensive mechanism capable of achieving real-time path optimization, feasibility verification, and self-recovery under dynamic fluidic constraints. When droplets encounter blockages or collisions during operation, current systems often fail to recover promptly, leading to task interruption or testing failure. This limitation severely constrains the application potential of intelligent optimization algorithms and parallel testing technologies in practical DMFB online testing platforms.

To address the aforementioned challenges, this paper proposes a hybrid optimization method based on the physical characteristics of DMFBs and an improved Sparrow Search Algorithm. On the standard 7 × 7–15 × 15 DMFB benchmark chips, the proposed method yields offline test paths whose lengths are equal to the Euler path length ($N_{EC}-2$), indicating that, for these regular layouts, the shortest test paths have reached the known optimal value, while the overall path stability is also effectively enhanced. The main contributions of this study are summarized as follows:

- A “priority encoding–path decoding” framework is proposed for DMFB test path optimization, which transforms the test path planning into a search problem within a priority parameter space. This framework fully leverages the electrode network structure of the DMFBs and abstracts the droplet traversal process into a graph-theoretic model, thereby enabling a rational integration of test path decision-making and fluidic constraint handling. As a result, it effectively reduces the complexity of path planning under intricate fluidic constraints and significantly enhances the search efficiency for optimal test paths.
- A deeply optimized Improved Sparrow Search Algorithm (ISSA) is developed to address the high-dimensional and strongly constrained search space of DMFB test path planning. Tent chaotic initialization is employed to enhance population diversity within the two-dimensional chip layout, while cosine adaptive weights and Elite Opposition-based Learning (EOBL) are used to balance global exploration with local exploitation. In addition, a Gaussian perturbation mechanism is incorporated to help the algorithm escape local optima, thereby improving convergence speed and solution quality for large-scale DMFBs.
- A robust path generation and rescue mechanism for online testing is established to dynamically adjust the test droplet route in real time when experimental droplets occupy the workspace. When the detection path becomes blocked, feasibility verification and short-range graph search are automatically triggered, ensuring continuous and conflict-free path execution. This mechanism effectively enhances the reliability and execution stability of DMFB path planning in dynamic testing scenarios.

## 2. DMFB Test Model

To effectively address the test path optimization problem in DMFBs, the problem must first be formally defined and mathematically modeled accurately. This chapter introduces and analyzes the testing principle of DMFBs, abstracts the physical chip structure into a graph-theoretic model, defines the key fluidic constraints that must be considered in online testing, and ultimately formulates the overall problem as a constrained combinatorial optimization objective.

### 2.1. DMFB Structure and Droplet Driving Principle

**Figure 1:**
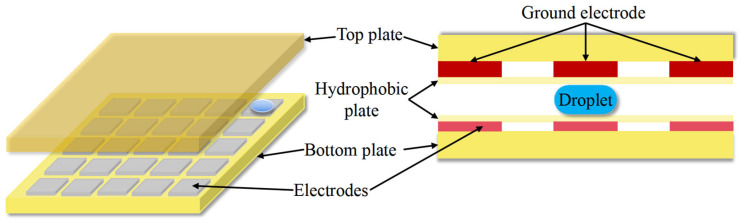
DMFB Structure.

A typical sealed DMFBs adopt a “sandwich” structure, consisting a hydrophobic layer and a dielectric layer positioned between two parallel electrode plates. The top plate is made of transparent conductive glass, while the bottom layer comprises a programmable two-dimensional electrode array, as shown in Figure 1. By applying time-sequenced control voltages to the lower control electrode array, the contact angle between the droplet and the electrode surface is modulated, creating a surface energy gradient that drives the droplet to move from one electrode unit to the adjacent unit. Consequently, droplet dispensing, merging, mixing and transportation can be precisely controlled at the micro-and nanoscales [17,23].

The droplet motion is governed by the electrowetting-on-dielectric (EWOD) effect, which can be described by the Young–Lippmann equation relating the contact angle to the applied voltage. In this work, we follow the common abstraction adopted in DMFB CAD research [8,10]: each actuation cycle moves a droplet by at most one electrode pitch, and the detailed fluid dynamics are not explicitly simulated. Instead, the fluidic feasibility is enforced by the static and dynamic spacing constraints in Equations (4) and (5).

### 2.2. Test Path Problem Description

**Figure 2:**
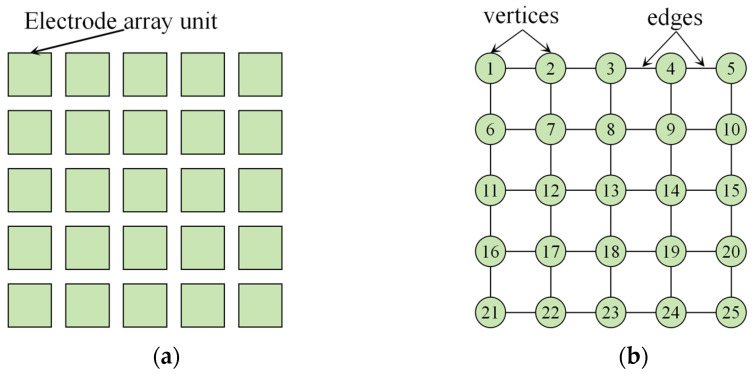
5 × 5 DMFB Chip Test Model. (**a**) Physical model of DMFB; (**b**) Graph theory model.

To detect catastrophic failures in DMFBs, such as electrode short circuits, open circuits or surface contamination, it is necessary to design a test droplet path that traverses the chip’s electrode array and cover all critical locations and interconnections. The DMFB can be abstracted as an undirected connected graph $G=\left(V,E\right)$, composed of nodes and edges, as shown in Figure 2. Here, $V=\left\{v_{1},v_{2},\ldots ,v_{mn}\right\}$ represents the set of electrode units in the chip, and $E=\left\{e_{ij}\right\}$ denotes the set of traversable edges between electrodes $v_{i}$ and $v_{j}$. As droplets move across the chip, its migration process can be represented as a traversal along the edges of this graph.

**Figure 3:**
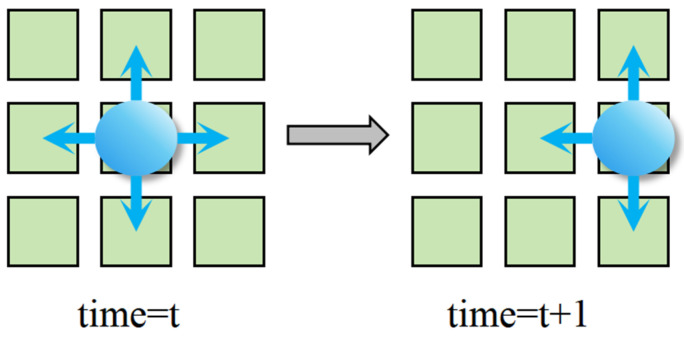
Schematic Diagram of Droplet Movement Direction.

During testing, the test droplet must systematically traverse the chip’s electrode to detect potential catastrophic faults. The movement of test droplets on the chip is governed by the EWOD actuation principle, which allows each droplet to move to one of its four neighboring electrodes (up, down, left, or right) in each discrete time step, as shown in Figure 3.

The test path of the test droplet is defined in Equation (1):

(1) $$Path=\left\{\left(v_{i_{1}},v_{i_{2}}\right),\left(v_{i_{2}},v_{i_{3}}\right),\ldots ,\left(v_{i_{k-1}},v_{i_{k}}\right)\right\}$$

where Path represents the movement path sequence of the test droplet on the chip, which can be regarded as an ordered sequence of edges on the graph $G=\left(V,E\right)$. Each pair of adjacent nodes $\left(v_{i_{j}},v_{i_{j+1}}\right)$ represents the movement of the droplet from node $v_{i_{j}}$ to adjacent nodes $v_{i_{j+1}}$ at the $j^{th}$ time step, corresponding physically to one movement of the droplet transition between adjacent electrodes. The index sequence $\left(i_{1},i_{2},\ldots ,i_{k}\right)$ represents the visiting order of nodes; thus, the construction of the test path is essentially a node visiting order optimization problem.

From an implementation point of view, the discrete path in Equation (1) is translated into a sequence of electrode actuation patterns on the physical DMFB. According to Equation (1), the path is represented as an ordered sequence of edges $\left(v_{i_{1}},v_{i_{2}}\right),\left(v_{i_{2}},v_{i_{3}}\right),\ldots ,\left(v_{i_{k-1}},v_{i_{k}}\right)$. At the *j*-th time step, which corresponds to traversing edge $\left(v_{i_{j}},v_{i_{j+1}}\right)$, the controller biases the electrode at node $v_{i_{j+1}}$ to the actuation voltage and releases the electrode at node $v_{i_{j}}$ to the idle state, while all non-participating electrodes remain at their default potential. Under the standard EWOD actuation regime, we assume that a droplet can reliably complete one hop between adjacent electrodes within one time step, so that the path length (i.e., the number of edges in the path) is directly proportional to the total actuation time.

### 2.3. Offline Test Model

Offline testing is a fundamental model of DMFBs testing, typically performed during the chip fabrication stage to detect static electrode faults. In this mode, the test droplet traverses the electrode array independently, without interference from experimental droplets, and the path planning only needs to satisfy geometric reachability.

To achieve comprehensive detection of all electrode connection edges, the offline test path must traverse every inter-electrode edge at least once. This requirement can be naturally modeled by a Eulerian circuit or a Eulerian path on the graph representation of the DMFB. In graph theory, a path is defined as a Eulerian path if it passes through every edge of the graph exactly once. If the starting point and ending point of the path coincide, it is referred to as a Eulerian circuit. A Eulerian path exists if and only if the graph has exactly 0 or 2 vertices with odd degree. If all vertex degrees are even, the graph admits an Eulerian circuit.

**Figure 4:**
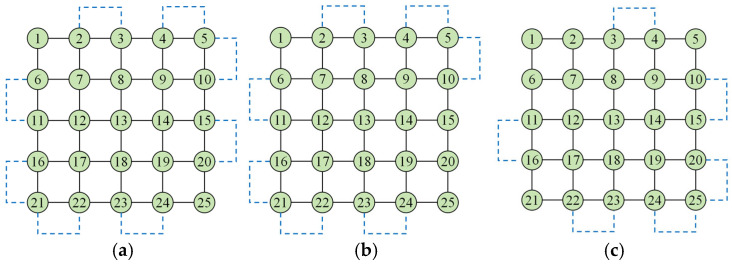
Eulerian Loop and Eulerian Path. (**a**) Eulerian circuit model; (**b**) Eulerian circuit model 1; (**c**) Eulerian circuit model 2.

When the original graph G does not admit a Eulerian circuit, it can be transformed into a new graph G′ with a Eulerian circuit by adding supplementary (virtual) edges to convert all odd-degree vertices into even-degree ones. A schematic of the Eulerian circuit model is shown in Figure 4a, and the corresponding Eulerian path models are shown in Figure 4b,c. Figure 4b contains one fewer edge than the Eulerian circuit, while Figure 4c contains two fewer edges than the Eulerian circuit.

In the DMFB testing problem, the number of edges in the Eulerian path directly reflects the minimum number of traversal steps required to cover all connected edges. Assume that the test chip model is a two-dimensional m×n electrode array, the number of nodes and edges are given by V=m×n and $N_{E}=m\left(n-1\right)+n\left(m-1\right)$, respectively. According to reference [10], for such a regular array, the number of edges of the Eulerian circuit is $N_{EC}$, as shown in Equation (2):

(2) $$N_{EC}=\left\{\begin{array}{c} 2mn-4,\;\mathrm{When}\;\mathrm{both}\;m\;\mathrm{and}\;n\;\mathrm{are}\;\mathrm{even}\\ 2mn-2,\;\mathrm{other} \end{array}\right.$$

which distinguishes the parity of the array dimensions. As illustrated in Figure 4b,c, when two distinct vertices are chosen as the start and end points, the corresponding Eulerian paths contain one or two fewer edges than the Eulerian circuit, i.e., their lengths are $N_{EC}-1$ and $N_{EC}-2$, respectively.

For such regular m×n layouts under the single-test-droplet offline model, these Eulerian paths require the minimum possible number of edge traversals to cover all inter-electrode connections. Thus, the Euler path length (i.e., $N_{EC}-1$ or $N_{EC}-2$, depending on the start and end locations) is adopted in this work as the theoretical optimal value of the offline test path length on the benchmark chips. In other words, when the shortest offline test path obtained by a given algorithm has a length of $N_{EC}-1$ or $N_{EC}-2$ on these regular arrays, we say that the optimal test path has been reached for this specific layout.

In the general formulation of the offline test path planning problem, if the starting point and ending point of the path coincide, the theoretical optimal length equals the Eulerian-circuit length $N_{EC}$; If the starting point and ending point of the path do not coincide, the theoretical optimal length equals the Eulerian-path length $N_{EC}-1$ or $N_{EC}-2$, as discussed above. The objective of offline test path planning is therefore to minimize the total length of the test path while ensuring that all edges are visited at least once. The corresponding objective function can be written as in Equation (3), where the cost of moving the droplet between two adjacent electrodes is generally taken as 1.

(3) $$minL=\underset{\left(v_{i},v_{j}\right)\in P}{\sum }c\left(v_{i},v_{j}\right)$$

Here, $c\left(v_{i},v_{j}\right)$ is the length cost of the droplet moving between adjacent electrodes, which is generally taken as 1.

### 2.4. Online Test Model

Unlike offline testing, online testing requires that test droplets coexist with experimental droplets, which perform biochemical experiments on the DMFB. Both types of droplets execute their respective tasks simultaneously, thereby enabling the detection of dynamic faults such as electrode degradation and time-varying short circuits during operation. In this case, if the test droplet and the experimental droplet occupy directly adjacent or diagonally adjacent electrode, they may merge with each other, resulting in experimental abnormalities or even complete failure. Therefore, fluidic constraints must be considered in the online testing.

Let $R_{t}^{i}$ and $C_{t}^{i}$ denote the row and column positions of droplet *i* at time *t*, respectively, and let $R_{t}^{j}$ and $C_{t}^{j}$ represent the corresponding positions of droplet *j* at the same time. To prevent undesired merging or collisions between test droplets and experimental droplets during movement, spatial constraints must be imposed on their relative positions. Depending on the droplet state, fluidic constraints can be categorized into static or dynamic type, which can be mathematically expressed as follows:

1. Static Constraint

When two droplets are stationary at the same time, the differences in their row or column coordinates within the electrode array should be at least 2. This avoids direct adjacency or diagonal contact between droplets and thereby prevents unintended coalescence. The constraint can be expressed as follows:

(4) $$\left|R_{t}^{i}-R_{t}^{j}\right|>1\;or\;\left|C_{t}^{i}-C_{t}^{j}\right|>1$$

Equation (4) indicates that, at any given time *t*, if two droplets are simultaneously stationary, their center electrodes must be separated by at least one unit in either the row or column direction. Violation of this condition will cause the droplets coalescence, resulting in experimental failure.

2. Dynamic Constraint

When droplets are in motion, it is also necessary to prevent them from occupying the same or crossing electrodes in adjacent time steps, in order to avoid collisions or overlap. The dynamic constraints can be expressed as follows:

(5) $$\left|R_{t}^{i}-R_{t+1}^{j}\right|>1\;or\;\left|R_{t+1}^{i}-R_{t}^{j}\right|>1\;or\;\left|C_{t}^{i}-C_{t+1}^{j}\right|>1\;or\;\left|C_{t+1}^{i}-C_{t}^{j}\right|>1$$

Equation (5) defines the relative positional constraints of droplets between consecutive time steps, ensuring that the motion trajectories of droplets *i* and *j* do not cross or overlap. Otherwise, collisions or merging will occur during their movement.

**Figure 5:**
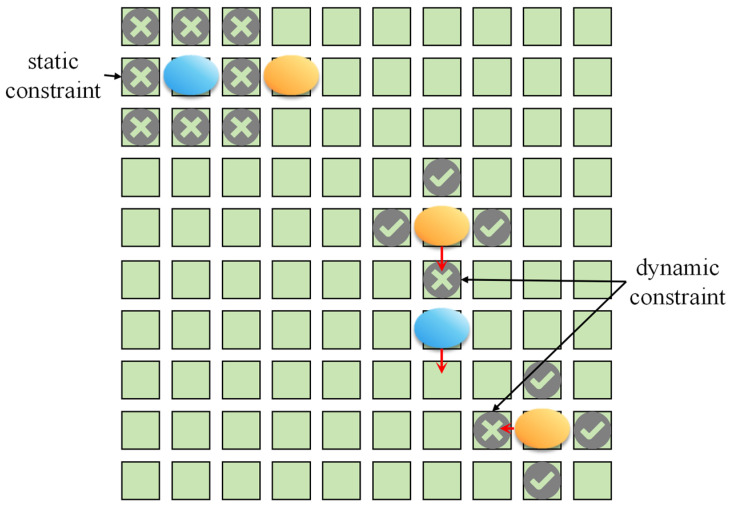
Schematic diagram of fluidic constraints.

Together, the static and dynamic constraints define the safe operation space for droplet motion on the DMFBs. The combination of these two constraints ensures that the test sequences generated by the path planning algorithm satisfy physical fluidic feasibility in both spatial and temporal dimensions. A schematic diagram of static and dynamic fluidic constraints is shown in Figure 5, where the cross marks denote cells that the blue droplet cannot reach in the next time step, while the tick marks indicate reachable cells.

During the online testing process of DMFBs, fluidic constraints significantly increase the complexity of the test path planning problem. To avoid conflicts between the test droplets and experimental droplets, a tabu matrix is constructed based on the movement trajectories of the experimental droplets, ensuring compliance with static constraint conditions. This matrix records the electrode positions that the test droplet is prohibited from accessing at each time step, thereby being used to guide the feasibility assessment of the test path.

During the movement of the test droplet, the system determines in real time whether its next candidate cell is included in the tabu matrix corresponding to the current time step, thereby verifying whether the static constraint conditions are satisfied. For dynamic constraints, the tabu matrix entries of the corresponding cell at adjacent time steps must also be checked to ensure that the test droplets do not cross or exchange positions with the trajectories of the experimental droplets. Only candidate cells that simultaneously satisfy both static and dynamic constraints are considered valid for droplet movement.

Because test droplets must avoid the dynamic path of the experimental droplets during path planning, it may be necessary at certain time steps to wait (to maintain a safe margin) or detour (to bypass occupied regions). Such dynamic obstacle avoidance behavior significantly increases the complexity of online test path planning, making it more challenging to achieve the optimal testing efficiency while maintaining operational safety.

In contrast to offline testing, online testing introduces additional time-varying fluidic constraints, under which some edges may be temporarily forbidden at certain time slots. As a result, a Eulerian path that attains the offline lower bound $N_{EC}-1$ or $N_{EC}-2$ may no longer be feasible. Therefore, in the online experiments, $N_{EC}$ and its related Euler path lengths are only used as geometric reference bounds rather than as guaranteed optimal values, and the performance of different algorithms is evaluated by comparing the lengths and stability of the feasible test paths they obtain under the imposed fluidic constraints.

## 3. Hybrid Algorithm Structure Based on Improved Sparrow Search Algorithm and Priority Strategy

To address the complex online test path optimization problem defined in Chapter 2, this study proposes a novel hybrid intelligent optimization method. In the abstracted chip graph model, each edge is assigned a randomly initialized priority coefficient that determines the traversal order of droplets during testing. Based on the initial priority sequence, the system generates an initial traversal path that satisfies the fluidic constraint. Subsequently, the intelligent optimization algorithm performs global search and reorganization of the priority coefficients, continuously guides the path generator toward more optimal solutions. Through iteratively optimizing the priority sequence, the system ultimately obtains a shorter test path along with the corresponding electrode activation sequence, thereby achieving global optimization of test efficiency.

### 3.1. Priority Strategy Design

In DMFB testing, the path of droplet motion is not only constrained by electrode actuation and spatial topology but must also satisfy temporal sequencing and fluidic safety constraints. To address these complexities, a priority-based path generation mechanism is proposed. By introducing a “priority encoding–path decoding” framework, the proposed method ensures both certainty and controllability in the path generation process. This section elaborates on the principles of priority encoding, the priority-based path generation procedure, and the intelligent rescue strategy.

#### 3.1.1. Principles of Priority Encoding

To enable efficient control of the path planning process through intelligent optimization algorithms, this paper introduces a priority-based encoding mechanism that abstracts the complex behavior of path generation into a set of continuously adjustable control parameters. Specifically, the system assigns a real-valued priority coefficient to each edge and constructs the following matrix:

(6) $$P=\left[p_{1},p_{2},\ldots ,p_{E_{num}}\right]$$

where $E_{num}$ denotes the total number of edges, and $p_{i}\in \left[0,1\right]$ represents the traversal priority of edge $e_{i}$ during path generation. A larger $p_{i}$ value indicates that the corresponding edge is more likely to be selected by the droplet.

**Figure 6:**
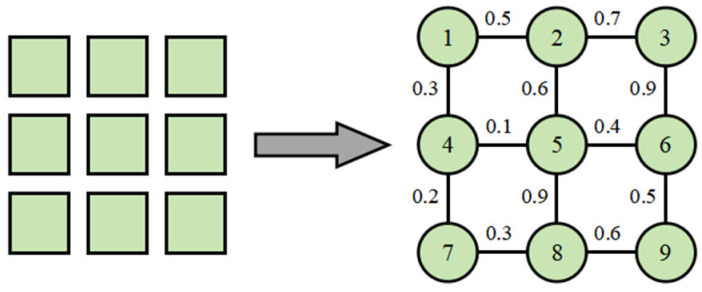
Schematic Diagram of Priority in DMFB Graph Model.

The schematic diagram of the priority encoding mechanism is shown in Figure 6. The advantage of this priority encoding approach lies in its ability to construct a continuously optimizable parameter space for path generation, rather than directly defining a complete path sequence. By adjusting the distribution of the priority vector P, the system can efficiently generate diverse feasible paths under constraints, providing a robust foundation for intelligent algorithm optimization process.

#### 3.1.2. Priority-Based Offline Test Path Generation Mechanism

The offline testing mode is applicable for chip factory testing or static performance verification stages. It is characterized by the existence of no dynamic interactions between droplets, with constraints determined solely by the chip’s topological structure. In this mode, the test droplet not need to consider the trajectories of experimental droplets but only needs to traverse all electrode connection edges. Let the current droplet’s node be $v_{i}$, and its set of neighboring nodes be defined as follows:

(7) $$N\left(v_{i}\right)=\left\{v_{j}|\left(v_{i},v_{j}\right)\in E\right\}$$

The system then filters out the set of unvisited edges from all neighboring nodes as follows:

(8) $$E_{unvisited}\left(v_{i}\right)=\left\{e_{ij}|e_{ij}\notin E_{visited}\right\}$$

The optimal movement direction is then selected based on priority coefficients as follows:

(9) $$v_{next}=\arg max_{v_{j}\in N\left(v_{i}\right)}p\left(e_{ij}\right),e_{ij}\notin E_{visited}$$

This iterative process continues until all edges have been traversed. Furthermore, during path generation, if all adjacent edges of the current node have been visited, the system performs a global search to identify for the endpoints of all unvisited edges and selects the nearest candidate node to the current position using a breadth-first search (BFS) algorithm.

#### 3.1.3. Priority-Based Online Test Path Generation Mechanism

Unlike offline mode, the online testing mode requires real-time path planning during experiment execution. At this stage, test droplet and experimental droplet share chip resources, requiring strict compliance with fluidic safety and spatiotemporal synchronization constraints. Accordingly, a tabu list is constructed to record electrode units that are inaccessible at each time step. In the present work, the spatiotemporal trajectories of all experimental droplets are assumed to be pre-scheduled by a front-end assay synthesis tool and remain fixed during testing; the tabu list is therefore constructed offline from this deterministic schedule and then queried online during test path generation. At time *t*, the tabu list is defined as follows:

(10) $$Tabu\left(t\right)=\left\{v_{k}\in V|v_{k}\;is\;occupied\;by\;experimental\;droplets\right\}$$

During path expansion, the system filters all neighboring nodes that satisfy the security conditions:

(11) $$N_{valid}\left(v_{i},t\right)=\left\{v_{j}\in N\left(v_{i}\right)|v_{j}\notin Tabu\left(t-1\right),Tabu\left(t\right),Tabu\left(t+1\right)\right\}$$

When multiple candidate directions exist, the algorithm selects the edge with the highest priority for the next move:

(12) $$v_{next}=\arg max_{v_{j}\in N_{valid}\left(v_{i},t\right)}p\left(e_{ij}\right),e_{ij}\notin E_{visited}$$

When $N_{valid}\left(v_{i},t\right)=\emptyset$, it indicates that the test droplet has no safe feasible path at the current time step.

If the test droplet cannot find any adjacent node satisfying the constraints at a given time step, it enters a dynamic deadlock state. In previous studies, typical recovery strategies include simple backtracking or random relocation. While these methods are straightforward to implement, they suffer from two limitations: first, they lack globality, attempting path recovery only within a local scope; second, they lack spatiotemporal feasibility analysis, easily causing new path conflicts or droplet overlaps. To address this limitation, this paper proposes an intelligent rescue mechanism with globality and spatiotemporal validation capability to adaptively reconstruct droplet paths when dynamic deadlock occur. The mechanism integrates shortest path search from graph theory with tabu-based constraint evaluation. This ensures path connectivity restoration at minimal cost while maintaining droplet movement safety, enabling continuous execution of testing tasks in dynamic environments.

Specifically, when the test droplet enters a dynamic deadlock, the algorithm activates the intelligent rescue mechanism. First, the system extracts potential target nodes from all endpoints of unvisited edges to construct a candidate target set:

(13) $$C=\left\{v_{1,}v_{2,}\ldots v_{k,}\right\}$$

Subsequently, based on the undirected graph model *G* = *V*, *E*, the geometric shortest length from the current node *vcur* to each candidate target is computed as Equation (14), and sort candidate nodes by distance, and the nearest feasible target is prioritized.

(14) $$L\left(v_{i}\right)=len\left(shortestpath\left(G,v_{cur},v_{i}\right)\right)$$

For each candidate rescue path $path_{rescue}$, the algorithm performs spatiotemporal feasibility verification. If any node along the path is marked as forbidden in the tabu list at its corresponding future time step, the path is discarded. Only when paths satisfy the follow condition are considered feasible and executed.

(15) $$\forall t_{j}\in \left[t_{cur},t_{cur}+\left|path_{rescue}\right|-1,\right],\;Tabu\left(t\right)=0$$

**Figure 7:**
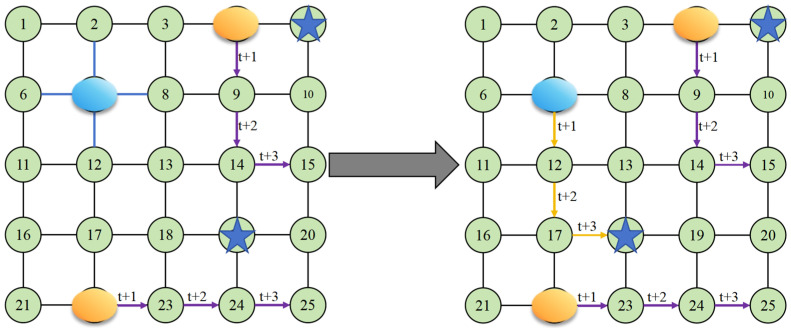
Rescue Mechanism Schematic Diagram.

As the droplet traverses the rescue path, the algorithm marks corresponding edges as visited and updates the path and time indices to ensure traversal continuity and temporal consistency. The overall rescue mechanism is illustrated in Figure 7.

In Figure 7, blue edges represent visited edges, blue droplets denote test droplets, yellow droplets indicate experimental droplets, and star-shaped icons mark the candidate target points. The left subfigure illustrates a scenario in which the test droplet reaches a state where all adjacent edges have already been visited. The path indicated by the purple arrows represents the edges and nodes that the experimental droplet will traverse over the next three time steps. At this stage, the system selects the shortest feasible path that satisfies the fluidic constraints and leads to the nearest candidate target point. The yellow arrow in the right subfigure depicts this optimal rescue path, after which the online testing process continues.

From a computational perspective, the core operation of the rescue mechanism is the shortest path search on the chip’s graph model. Using a standard breadth-first search (BFS), the time complexity of each shortest path query is *O* (|*V*| + |*E*|), where |*V|* = *m* × *n* is the number of electrodes and |*E*| ≈ 2 *mn* is the number of edges. For the largest chip size considered in this study (15 × 15), |*V*| = 225. The feasibility check for each step along the candidate path involves a constant-time lookup *O* (1) in the time-indexed tabu matrix. Therefore, the computational cost of the rescue process is modest compared with the droplet actuation interval (on the order of hundreds of milliseconds in typical DMFB systems) and remains acceptable for real-time online applications on the benchmark sizes considered.

Operationally, the priority-based online path generator runs in a supervisory controller that is synchronized with the DMFB actuation clock. At each time step, the controller uses the current droplet positions and the time-indexed tabu matrix to decide the next cell for the test droplet, optionally invoking the rescue mechanism when a dynamic deadlock is detected. The resulting next position is then translated into an electrode actuation pattern and issued to the on-chip driver circuitry, so that the planned path and the physical droplet motion remain tightly coupled in real time.

### 3.2. The Design of Improved Sparrow Search Algorithm

**Figure 8:**
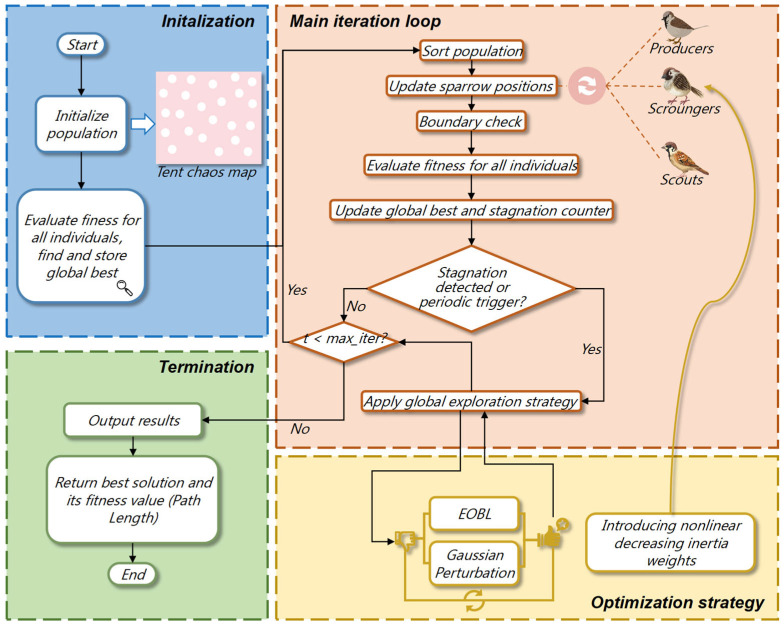
ISSA Flowchart.

In the aforementioned priority-based path generation mechanism, the quality of test paths heavily depends on the distribution of priority parameters. However, within the high-dimensional and strongly constrained DMFB testing space, efficiently searching parameters that generate optimal path sequences becomes critical to algorithmic performance. To further enhance the global search capability and convergence stability of the priority-based path generation mechanism, this paper proposes an Improved Sparrow Search Algorithm (ISSA). Functioning as an optimizer, the ISSA guides the path generator to produce superior test paths though global exploration of the priority parameter set. Building upon the traditional Sparrow Search Algorithm (SSA) [24], the proposed method integrates Tent chaotic initialization, cosine adaptive weighting, EOBL, and Gaussian random perturbation mechanisms. These enhancements collectively improve the algorithm’s convergence speed, stability, and global search performance under complex dynamic constraint scenarios. Figure 8 shows the flowchart of the proposed algorithm.

#### 3.2.1. Population Initialization and Encoding Method

In the ISSA, the path optimization problem is transformed into an optimization problem for priority parameters. Each sparrow individual represents a potential solution as a real-valued vector, where each component corresponds to the priority coefficient of an edge in the graph. That is, each individual encodes a complete priority allocation scheme. This encoding scheme is decoded by the path generator module described in the previous section into a complete test path that satisfies both electrode activation constraints and droplet avoidance constraints. The total path length serves as the fitness value for that individual. Therefore, the core objective of the optimization process is to search globally for the optimal priority vector, aiming to obtain the shortest droplet test path that satisfies all constraints.

Specifically, let the population size be *N*; the position vector of each individual is defined as follows:

(16) $$X_{i}=\left[p_{1},p_{2},\ldots ,p_{d}\right],\;i=1,2,\ldots ,N$$

Here, *d* denotes the number of edges $E_{num}$ in the chip graph model, and each priority coefficient satisfies $p_{j}\in \left[0,1\right]$.

The individual fitness function is defined as follows:

(17) $$f\left(X_{i}\right)=L\left(Path\left(X_{i}\right)\right)$$

where $L\left(Path\left(X_{i}\right)\right)$ denotes the path length generated according to the priority sequence $X_{i}$. The objective of the algorithm is to minimize this fitness function:

(18) $$minf\left(X_{i}\right)=minL\left(Path\left(X_{i}\right)\right)$$

This mechanism implements a closed-loop mapping of “priority optimization–path generation–path evaluation”, thereby providing a clear search objective for the ISSA.

#### 3.2.2. Chaotic Population Initialization

The traditional SSA employs a random initialization method, which frequently results in an uneven distribution of initial individuals, thereby reducing the algorithm’s global search efficiency. To overcome this issue, the ISSA introduces the Tent chaotic map for population initialization, as defined in Equation (19). This approach enhances the population’s diversity and spatial coverage at the early search stage.

(19) $$x_{n+1}=\left\{\begin{array}{c} \frac{x_{n}}{a},x_{n}<a\;\\ \frac{1-x_{n}}{1-a},\;x_{n}\geq a \end{array}\right.$$

Here, $a\in \left(0,1\right)$ is a control parameter, set to 0.7 in this study. Through the iteration, a chaotic sequence $\left\{x_{n}\right\}$ with strong exploration capability is obtained, which is then assigned to the priority parameters of each individual to construct a more uniform initial search population.

#### 3.2.3. Core Update Mechanism and Adaptive Weights

The core search process of the ISSA retains the “producer-scrounger-scout” social hierarchy from the SSA but introduces a cosine adaptive weight mechanism during the scrounger phase to dynamically balance global exploration and local exploitation.

1. Producer Update Strategy

The producer is responsible for global exploration, and its position update strategy follows the standard SSA, as defined in Equation (20):

(20) $$X_{i,j}^{t+1}=\left\{\begin{array}{c} X_{i,j}^{t}\cdot e^{-\frac{i}{\alpha T_{max}}},\;R_{2}<ST\\ X_{best,j}^{t}+Q\cdot L,R_{2}\geq ST\; \end{array}\right.$$

where $X_{i,j}^{t+1}$ denotes the position of the $i^{th}$ sparrow in the $j^{th}$ dimension at iteration *t* + 1. $T_{max}$ represents the maximum number or iterations, and α is a random number in the interval (0,1], set to 0.7 in this study. $R_{2}\in \left[0,1\right]$ is the early-warning threshold, and $ST\in \left[0.5,1\right]$ is the safety threshold, set to 0.8 in this study. *Q* is a random number following a standard normal distribution. $X_{best,j}^{t}$ denotes the current global optimal position. *L* is a 1 × dim matrix of ones.

2. Scrounger Adaptive Strategy

For a scrounger with high fitness rankings, this paper introduces a nonlinearly decreasing inertial weight, and its position update formula is as follows:

(21) $$X_{i,j}^{t+1}=w\left(t\right)\cdot X_{i,j}^{t}+\left(1-w\left(t\right)\right)\cdot (X_{best,j}^{t}+\left|X_{i,j}^{t}-X_{best,j}^{t}\right|\cdot N\left(0,1\right))$$

Here, $w\left(t\right)$ is a nonlinear inertia weight that varies with the number of iterations *t*, and $w\left(t\right)$ is calculated as follows:

(22) $$w\left(t\right)=0.5\cdot \left(1+\cos(\frac{t}{T_{max}}\cdot \pi ,)\right)$$

For a scrounger with lower fitness rankings, the update method follows the standard SSA:

(23) $$X_{i,j}^{t+1}=Q\cdot e^{\frac{X_{worst,j}^{t}-X_{i,j}^{t}}{i^{2}}}$$

where $X_{worst,j}^{t}$ is the current global worst position, and $N\left(0,1\right)$ is a random number following a standard normal distribution.

3. Scout Update Strategy

The update strategy of the scout remains consistent with the standard SSA, as shown in Equation (24):

(24) $$X_{i,j}^{t+1}=\left\{\begin{array}{c} X_{best,j}^{t}+\beta \cdot \left|X_{i,j}^{t}-X_{best,j}^{t}\right|,f_{i}>f_{g}\\ X_{i,j}^{t}+K\cdot \left(\frac{\left|X_{i,j}^{t}-X_{worst,j}^{t}\right|}{(f_{i}-f_{w})+\epsilon }\right),\;f_{i}=f_{g}\; \end{array}\right.$$

where β is the step-size control parameter following a normal distribution, $K\in \left[-1,1\right]$ is a random number, and $f_{i}$, $f_{g}$, and $f_{w}$ represent the fitness values of the current, global best, and the global worst individuals, respectively. ϵ is an extremely small constant to prevent the denominator from becoming zero.

#### 3.2.4. Stagnation Detection and Hybrid Breakout Strategy

A common challenge in the standard SSA is premature convergence to local optima during the later iterations, caused by a decline in population diversity. To mitigate this issue, a hybrid global exploration mechanism combining EOBL and Gaussian perturbation is introduced to enhance the algorithm’s ability to escape local extrema.

1. Stagnation Escape Mechanism Based on EBOL

EBOL is an efficient global search strategy inspired by a heuristic assumption: The opposite position of a high-quality solution also has a high probability of being near an even better solution. In the ISSA, EOBL is designed as a stagnation escape mechanism whose trigger condition is that the convergence process of the algorithm is stalled. In this work, convergence stagnation is detected by monitoring the global best fitness $f_{best}\left(t\right)$: if $f_{best}\left(t\right)$ does not improve for *S* consecutive generations (with *S* = 10) in our experiments), a stagnation event is declared and the EBOL-based escape strategy is activated. The formula is as follows:

(25) $$X_{best}^{\prime }\left(j\right)=a_{j}\left(t\right)+b_{j}\left(t\right)-X_{best}\left(j\right)$$

When stagnation is detected, the algorithm dynamically computes a search space based on the current population individual distribution $\left[a_{j}\left(t\right),b_{j}\left(t\right)\right]$, and we apply the above equation to the current elite individual $X_{best}\left(j\right)$ to generate its reverse solution $X_{best}^{\prime }\left(j\right)$. This operation is equivalent to performing a large-scale and purposeful jump in the search space to explore the unknown region opposite to the current optimal region, and thus provides an efficient way for the algorithm to escape from local optima.

2. Local Fine Search and Diversity Injection with Gaussian Perturbation

To periodically improve the population vitality when the algorithm is not stagnating, this paper also introduces the Gaussian perturbation mechanism. This strategy is activated at fixed iteration intervals, and a random disturbance conforming to a normal distribution is applied to the current optimal solution, as shown in the formula:

(26) $$X_{best}^{\prime \prime }\left(j\right)=X_{best}\left(j\right)+\lambda \cdot \left(ub_{j}-lb_{j}\right)\cdot N\left(0,1\right)$$

In a preset iteration period, the algorithm will activate the local perturbation mechanism, and the above formula will be applied to the current elite individual $X_{best}\left(j\right)$ to generate its perturbation corresponding $X_{best}^{\prime \prime }\left(j\right)$. This operation can be viewed as a probabilistic probe from the current optimal point to the search space around it. Due to the centralization property of the standard normal distribution $N\left(0,1\right)$, the random value generated by it is close to zero with a large probability. Therefore, the perturbation in most cases is a small adjustment of the elite solution, which helps the algorithm to carry out finer local mining in the discovered dominant region, thereby enhancing its local development ability.

To provide a clear overview of the proposed method, the pseudocode for ISSA is shown in Algorithm 1.

**Algorithm 1** **Pseudocode for ISSA****Input:** Chip size m×n, Population size *N*, Max iterations $T_{max}$, Parameters $PD_{ratio}$, $SD_{ratio}$, $S_{T}$**Output:** Optimal priority vector $X_{best}$ and shortest path ${Path}_{best}$**Initialization:** Initialize population *X* using Tent chaotic map (Equation (19));              Calculate fitness $f\left(X_{i}\right)$ for all individuals using Path Generator;              Find global best $X_{best}$ and worst $X_{worst}$;              Set t=1, stagnation_counter=0;**While** ($t\leq T_{max}$) **do**  Sort population based on fitness values;  **For** each individual i=1 to *N* **do**   **If**
$i\leq N\times PD_{ratio}$
**then** // Producer     Update position $X_{i}^{t+1}$ using Equation (20);   **Else**     Update *w(t)* using cosine adaptive weight (Equation (22));       Update position $X_{i}^{t+1}$ using Equation (23);   **End if**  **End For**  Randomly select scouts and update positions using Equation (24);  Apply boundary control to all individuals;  Evaluate new fitness values $f\left(X_{i}^{t+1}\right)$;  Update global best $X_{best}^{new}$;  // Stagnation Detection and Breakout Strategy  **If**
$f\left(X_{best}^{new}\right)<f\left(X_{best}\right)$
**then**    $X_{best}\leftarrow X_{best}^{new},\;stagnation\_counter=0$;  **Else**    stagnation_counter←stagnation_counter+1  **End If**  **If**
$stagnation\_counter\geq S_{T}$
**then**Generate candidate $X_{EOBL}$ using EOBL (Equation (25));**If** $f\left(X_{EOBL}\right)<f\left(X_{worst}\right)$ **then** Replace $X_{worst}$ with $X_{EBOL}$;

stagnation_counter=0

  **End If**  **If** $t\left(mod\;20\right)==0$ **then**Generate candidate $X_{Gauss}$ using Gaussian Perturbation (Equation (26));**If** $f\left(X_{Gauss}\right)<f\left(X_{worst}\right)$ **then** Replace $X_{worst}$ with $X_{Gauss}$;  **End If**  t←t+1
**End while**
**Return** $X_{best},{Path}_{best}$

## 4. Experiment and Analysis

In this section, the test cases in references [10,12] are employed to perform both offline and online testing on five DMFBs of varying sizes (7 × 7, 9 × 9, 11 × 11, 13 × 13 and 15 × 15). The effectiveness of the proposed algorithm is verified by MATLAB R2024b simulation platform.

For the proposed ISSA, the key parameters are carefully configured and fine-tuned. The population size is set to 30 and the maximum number of iterations to 500 in both offline and online tests. The proportions of producers and scouts are set to 0.3 and 0.2, respectively, providing the population with sufficient exploratory capability in the high-dimensional search space. The stagnation threshold that triggers the EOBL-based escape mechanism is set to 10 generations, enabling a rapid response to convergence stagnation. In addition, Gaussian perturbations are activated every 20 generations with the perturbation coefficient fixed at 0.1, which periodically injects diversity into the population while avoiding excessive disturbance of the established convergence trend.

For the baseline algorithms (IGWO, PS-GA, IPSO, IACA and PMF), we follow the parameter settings reported in their original papers, and the corresponding numerical results in Table 1 and Table 2, Table 5 and Table 6 are directly quoted from those publications. The ISSA results, in contrast, are obtained from our own implementation on the same benchmark chips and under the same offline/online test models.

### 4.1. Offline Test Simulation

In this study, since no biochemical experiments were performed during the offline testing phase of the DMFB, only the scenario in which the test droplet traversed all electrode edges was considered. Table 1 presents the offline test results for chips of different sizes.

**Table 1:** Minimum path lengths in offline testing for different algorithms.

Chip Size	ISSA	IGWO [25]	PS-GA [16]	IPSO [14]	IACA [12]	PMF [10]
7 × 7	94	94	94	100	100	96
9 × 9	158	158	158	182	165	160
11 × 11	238	238	238	272	254	240
13 × 13	334	334	334	368	350	336
15 × 15	446	446	446	498	470	448

Table 1 reports the shortest offline test path lengths obtained by different algorithms on the five benchmark chips. The following observations can be made. (1) For all chip sizes, the proposed ISSA achieves the current best offline path length, which is identical to the results produced by IGWO and PS-GA and consistent with the known Euler path lower bounds on these regular arrays. When compared with IACA, IPSO and PMF, the proposed algorithm yields shorter paths, reducing the path length by up to 24, 52 and 2 edges, respectively. (2) As the chip size increases from 7 × 7 to 15 × 15, the absolute path length naturally grows for all algorithms, and the performance gap between ISSA and some baselines (particularly IPSO and IACA) becomes more pronounced. This indicates that the proposed algorithm maintains competitive performance and good robustness when addressing larger-scale and more complex DMFB test path instances. (3) Compared with other high-performance algorithms such as PMF, IGWO and PS-GA, ISSA produces equal or shorter path lengths across all chip sizes, suggesting stable solution quality and strong competitiveness in offline test path planning.

Although PMF, IGWO and PS-GA exhibit equivalent performance in path length, ISSA demonstrates distinct advantages in computational stability and convergence speed. Specific results are shown in Table 2, Figure 10 and Figure 11.

**Table 2:** Comparison of ISSA and IGWO algorithms in terms of stability of DMFB offline test path.

Algorithm	Chip Size	Minimum Value	Maximum Value	Mean Value	Standard Deviation
IGWO [25]	7 × 7	94	94	94	0
	9 × 9	158	158	158	0
	11 × 11	238	240	238	0.59
	13 × 13	334	337	335	1.69
	15 × 15	446	458	450	3.81
ISSA	7 × 7	94	94	94	0
	9 × 9	158	158	158	0
	11 × 11	238	240	238.08	0.34
	13 × 13	334	336	334.56	0.86
	15 × 15	446	454	449.80	2.12

Table 2 presents the detailed statistical results of ISSA and IGWO over 50 independent offline testing runs. The results show the following: (1) In terms of the shortest path length, both ISSA and IGWO exhibit essentially the same best performance, finding the same shortest path length across all chip sizes. (2) The standard deviation results indicate that for all fluctuating test cases (11 × 11 and larger), ISSA tends to be more stable than IGWO. Specifically, the standard deviations of ISSA are reduced by 42.37% and 49.11% for the 11 × 11 and 13 × 13 chips, respectively, and by 53.14% for the most challenging 15 × 15 chip. These observations suggest that ISSA produces a more concentrated distribution of high-quality solutions across multiple runs and is less sensitive to random fluctuations than a conventional swarm intelligence algorithm. (3) Regarding average performance and worst-case performance, the advantages of ISSA become more noticeable as chip size increases. In the tests on 13 × 13 and 15 × 15 chips, ISSA achieves lower mean and maximum path lengths than IGWO, indicating improved robustness on these larger and more complex problem instances.

**Figure 9:**
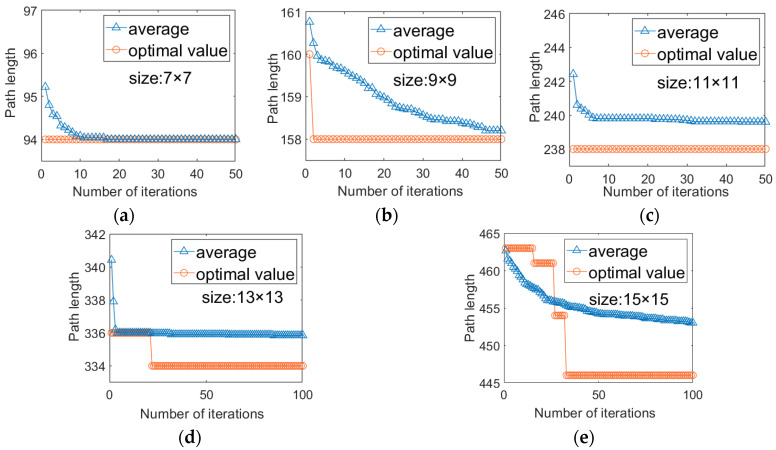
Offline Testing Convergence Curves of ISSA. (**a**) 7 × 7 array; (**b**) 9 × 9 array; (**c**) 11 × 11 array; (**d**) 13 × 13 array; (**e**) 15 × 15 array.

**Figure 10:**
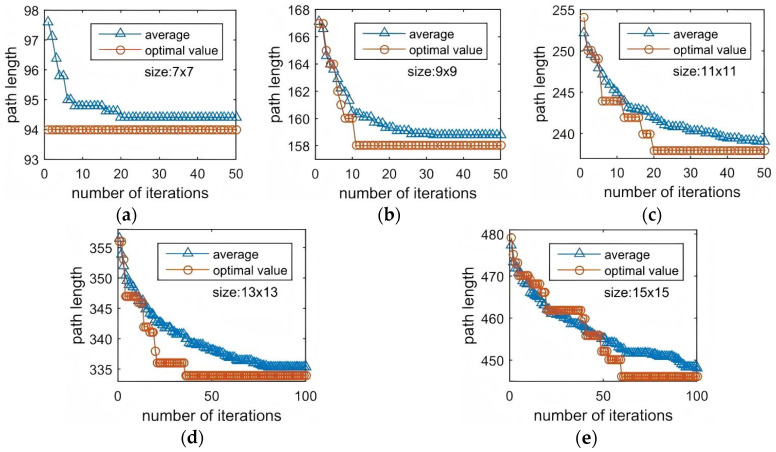
Offline Testing Convergence Curves of the PS-GA [16]. (**a**) 7 × 7 array; (**b**) 9 × 9 array; (**c**) 11 × 11 array; (**d**) 13 × 13 array; (**e**) 15 × 15 array.

To visually evaluate the convergence performance of the proposed ISSA, Figure 9 illustrates its convergence curves for chips of various sizes, while Figure 10 presents the convergence curves of PS-GA under the same conditions. In both figures, the blue triangle curve represents the average path length, reflecting the overall convergence trend, and the orange circle curve represents the optimal path length, reflecting the algorithm’s optimization capability.

From these figures, the following can be observed: (1) Across all the test cases, the optimal value curve of ISSA exhibits a remarkably steep initial descent, particularly for smaller chips ranging from 7 × 7 to 11 × 11. In most instances, the optimal solution is found within the very first iteration and remains unchanged thereafter. Even for medium and large chip such as 13 × 13 and 15 × 15, ISSA performs excellent convergence performance, typically reaching the optimal solution within 30 iterations. In contrast, PS-GA curves considerably more slowly. Except for the 9 × 9 size, where it initially found the optimal solution, all other sizes exhibited a step-like decrease process, with convergence speeds consistently lagging behind ISSA. These results clearly indicate that the introduction of the EBOL and Gaussian Perturbation strategies significantly enhances the algorithm’s ability to escape local optima, thereby improving convergence efficiency. (2) Furthermore, both optimal value and average convergence curves show that ISSA consistently achieves better initial solution quality compared to PS-GA at the beginning of the iterations. This advantage primarily arises from the Tent chaotic map initialization and the nonlinear inertial weighting strategies. These improvements provide the algorithm with a more uniformly distributed and higher-quality initial population, effectively balance the algorithm’s global exploration and local exploitation, enabling the entire population to converge toward advantageous regions more rapidly, which avoids redundant searches in invalid spaces, thereby improving the overall operational efficiency of the algorithm.

### 4.2. Online Test Simulation

After completing the offline simulations, this study further evaluates the optimization capability of ISSA in complex dynamic environments. To this end, two representative online biochemical experimental models are employed to conduct online testing of the DMFB. Unlike offline testing, online testing must simultaneously consider the dynamic motion of experimental droplets and their fluidic constraints with test droplets, thereby better reflecting the algorithm’s performance in scheduling and collision avoidance capabilities in practical applications.

**Figure 11:**
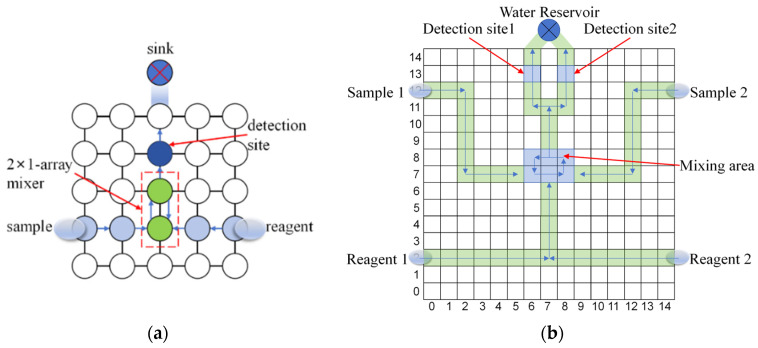
Schematic Diagram of Biochemical Experimental Model. (**a**) Model for a single experiment; (**b**) Model for a multiple experiment.

For small-size chips (such as 7 × 7, 9 × 9, 11 × 11, and 13 × 13 chip sizes), a single biochemical reaction process is adopted as the online testing model. As shown in Figure 11a, sample droplets and reagent droplets are released from the reservoir area, sequentially pass through a 2 × 1 mixing zone for complete reaction, undergo optical detection in the detection zone, and finally move to the waste reservoir. This workflow simulates a typical biochemical detection procedure on a DMFB. The temporal scheduling of experimental and test droplets achieved through time slots, as shown in Table 3.

For the large-scale 15 × 15 chip, a multi-biochemical parallel experiment model was adopted, as shown in Figure 11b. This model integrates two sets of sample-reagent reaction units that perform mixing, detection, and waste discharge operations at staggered time intervals to simulate the concurrent execution of multi-task biochemical reactions on the same chip. The corresponding experimental schedule table for the multi-biochemical parallel experiment is as Table 4:

These online test cases correspond to a predictive online testing scenario in which the assay scripts for all experimental droplets are fully determined in advance. The test path planner has access to this pre-scheduled information and uses it to construct a time-indexed tabu matrix that anticipates future resource conflicts. Truly fault-driven online testing with unexpected droplet delays, stochastic actuation failures, or dynamically appearing defects is beyond the scope of this work, and would require additional sensing and re-scheduling mechanisms on top of the current framework.

The parameter settings for online testing are kept consistent with those used in the offline testing. To evaluate the performance of the proposed ISSA in addressing online testing path problems, a comparative analysis with five representative algorithms—including IGWO, PS-GA, IPSO, IACA, and PMF—is presented in Table 5. The table reports the minimum path lengths achieved in the online testing phase. The experimental data in the table clearly show the following: (1) Across all five chip sizes, the proposed algorithm and PS-GA jointly obtain the shortest path lengths among the compared methods on these benchmarks. In contrast, classical algorithms such as IPSO, IACA and PMF yield noticeably longer paths. For the most challenging 15 × 15 chip, the path length obtained by ISSA is 17.9% shorter compared to IPSO, 5.3% shorter compared to IACA and 8.2% shorter compared to PMF, suggesting that ISSA can maintain relatively high solution quality in large-scale online test path planning compared with these baselines. (2) When compared with the more recent IGWO algorithm, ISSA achieves slightly shorter minimum path lengths across all chip sizes. Given that the absolute differences are small (typically within one or a few edges) and that the stopping criteria are not fully normalized across all methods, these results should be interpreted as indicating broadly comparable best-case performance, with a modest advantage for ISSA on the considered benchmarks. (3) As the chip size increases from 7 × 7 to 15 × 15, the relative performance gap between ISSA and algorithms such as IPSO, IACA and PMF becomes more pronounced. This trend indicates that ISSA remains competitive and robust as the problem scale and complexity grow, while the other classical algorithms deteriorate more noticeably in solution quality.

Overall, ISSA consistently achieves shorter path lengths and more stable performance outcomes in online testing path optimization, thereby confirming its modest but robust improvements for complex and dynamic DMFB scheduling scenarios.

**Table 5:** Minimum path length in online testing for different algorithms.

Chip Size	ISSA	IGWO [25]	PS-GA [16]	IPSO [14]	IACA [12]	PMF [10]
7 × 7	94	95	94	119	105	110
9 × 9	158	159	158	199	171	178
11 × 11	238	239	238	296	260	269
13 × 13	334	335	334	412	361	380
15 × 15	446	449	446	543	471	486

**Figure 12:**
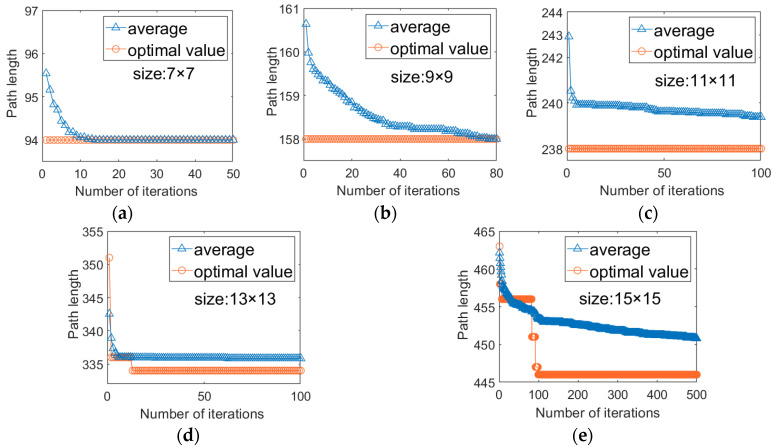
Online Testing Convergence Curves of ISSA. (**a**) 7 × 7 array; (**b**) 9 × 9 array; (**c**) 11 × 11 array; (**d**) 13 × 13 array; (**e**) 15 × 15 array.

**Figure 13:**
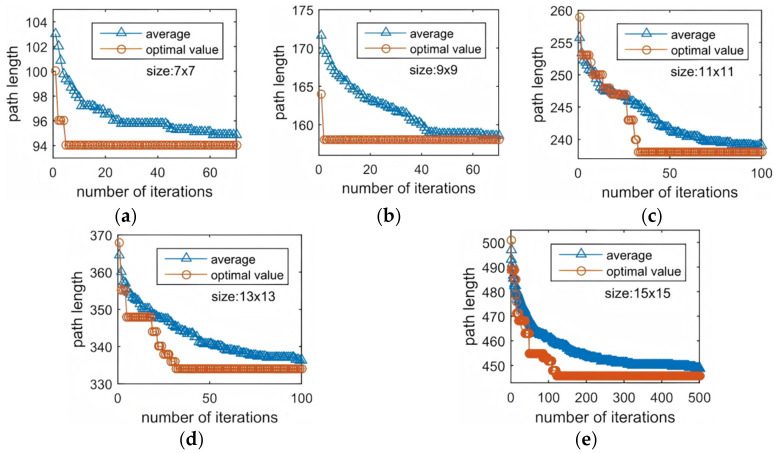
Ref. [16] Online Testing Convergence Curves of PS-GA. (**a**) 7 × 7 array; (**b**) 9 × 9 array; (**c**) 11 × 11 array; (**d**) 13 × 13 array; (**e**) 15 × 15 array.

To further assess the behavior of the proposed algorithm in a dynamic and constrained online testing environment, we carried out a comparative study against the IGWO and PS-GA on the same benchmark chips. This comparison focuses on the stability and adaptability of ISSA under time-varying fluidic conditions, as summarized in Table 6 and illustrated in Figure 12 and Figure 13.

As shown in Table 6, detailed statistical results for the ISSA and IGWO algorithms were obtained from 50 independent runs of online testing. Analysis of the data reveals the following: (1) Across all chip sizes, the minimum values obtained by ISSA are consistently lower than those achieved by IGWO. This demonstrates that even in dynamic online environments with tabu constraints, the proposed algorithm exhibits superior global optimization capabilities, consistently identifying higher-quality path solutions. (2) The standard deviation metric further confirmed ISSA’s remarkable advantage in optimization stability. Overall, ISSA exhibits lower standard deviation than IGWO across all chip sizes, reflecting less dispersion in search results and more stable convergence. Notably, for small chips as 7 × 7 and 9 × 9, ISSA achieves a standard deviation of zero, implying that the algorithm converges to the same optimal solution in every run. As chip sizes increase, minor fluctuations appear; however, ISSA’s standard deviation remains substantially lower than IGWO’s. Specifically, ISSA reduces the standard deviation by 77.12% and 53.01%, respectively. On the most complex 15 × 15 chip, its standard deviation of 2.08 represents a 39.4% reduction compared to IGWO’s 3.43. These results demonstrate that ISSA maintains high solution consistency even under complex dynamic constraints and high-dimensional search spaces. (3) In terms of average and maximum path lengths, ISSA also shows more favorable behavior. Its average path length remains below that of IGWO for all chip sizes, indicating better typical performance over multiple runs. Furthermore, the lower maximum path lengths imply that the worst-case outcomes of ISSA are generally better than those of IGWO, which is consistent with more robust convergence behavior.

Figure 12 and Figure 13 show the convergence curves of ISSA and PS-GA, respectively, with Figure 12 representing ISSA and Figure 13 representing the PS-GA. Analysis of the two sets of figures reveals the following: (1) A prominent difference lies in the convergence speed of two algorithms. In online testing of small-to-medium-sized chips ranging from 7 × 7 to 11 × 11, ISSA consistently finds the optimal value immediately at the start of the iterations. For the 13 × 13 chip, ISSA converges to the optimal value within 15 iterations and remains stable thereafter. In contrast, the PS-GA generally exhibits a “step-like” descent pattern during convergence, indicating that it frequently becomes trapped in local optima during the optimization process. On the most complex and challenging 15 × 15 chip, ISSA achieves the optimal value at the 99th iteration, whereas PS-GA reaches it only at the 120th iteration. This clearly demonstrates that the global exploration strategy embedded in ISSA enables faster convergence speed, more efficient escape from local optima, and accurate localization of the optimal solution under dynamic constraint environments. (2) The average convergence curves further confirm ISSA’s superior overall optimization performance. As shown in the figure, ISSA’s initial average value is significantly lower than PS-GA’s, with its convergence curve exhibiting a rapid declining during the early iterations before smoothly stabilizing near the mean. This behavior indicates that ISSA establishes a more favorable and well-distributed search space during initial population generation, which accelerates the overall convergence process.

Overall, ISSA clearly outperforms the PS-GA in terms of convergence speed, stability, and global optimization capability, exhibiting excellent efficiency and robustness across different chip sizes.

## 5. Discussion

The proposed DMFB test path planning method, which integrates priority decoding with ISSA, demonstrates significant advantages in both offline and online testing scenarios. The experimental results show that this approach achieves offline path lengths equal to the known Eulerian path on the benchmark chips and exhibits stronger robustness in convergence stability and overall optimization performance. Compared with existing studies, the proposed method performs better under complex electrode arrays and multiple constraint conditions, confirming the effectiveness of the hybrid strategy of “priority encoding + intelligent algorithm search + spatial–temporal possibility decoding”.

According to prior research on DMFB path planning, traditional approaches have primarily focused on heuristic algorithms and swarm intelligence algorithms. While these approaches improve optimization efficiency to some extent, they often become trapped in local optima when dealing with highly constrained and dynamic search spaces. In contrast, the proposed ISSA incorporates Tent chaotic mapping for initialization, a cosine adaptive convergence factor, EBOL, and a Gaussian perturbation mechanism. Together, these improvements achieve a superior balance between global exploration and local exploitation throughout the search process. Moreover, the introduced intelligent rescue mechanism effectively prevents redundant path extensions caused by traditional backtracking by integrating global connectivity evaluation and predictive tabu-region analysis across time steps.

It should be noted that although ISSA achieved strong stability and rapid convergence in simulation environments, droplet behavior in real DMFB hardware remains partially unpredictable due to actuation voltage variation and dielectric degradation. For instance, droplets maintain stable morphology within an actuation range of 50–70 Vpp; however, microbubble formation occurs when the voltage exceeds 80 Vpp, suggesting the onset of hydrolysis [26]. Therefore, current performance still requires further confirmation through hardware verification. Furthermore, this study primarily focuses on scenarios involving a single test droplet coexisting with a small number of experimental droplets. More complex scenarios, such as parallel scheduling of multiple test droplets and environments with faulty electrodes, remain open for future investigation.

Regarding the physical usage model of the chip, the proposed framework itself is agnostic to whether the DMFB is reused across multiple assay runs or treated as a disposable device. The offline test paths are naturally aligned with post-fabrication or pre-deployment screening, whereas the online testing and rescue mechanisms are designed to operate during assay execution to detect time-varying faults. In both reusable and single-use settings, the optimization strategy focuses on generating safe and efficient test paths under the given fluidic constraints, rather than on managing the physical lifetime of the chip.

In addition, the online simulations in this work assume deterministic, pre-planned trajectories for the experimental droplets and do not explicitly model unscheduled delays, stochastic actuation failures or time-varying defect activation. This setting is representative of design-time integration of testing with a known assay script, but it does not fully capture all sources of runtime uncertainty and congestion that may occur in practical DMFB platforms. Extending the proposed framework to incorporate runtime feedback, probabilistic disturbance models and highly congested multi-test-droplet scenarios will be an important direction for future research.

In summary, this study not only validates the performance advantages of ISSA in DMFB test path planning but also provides insights for future research on intelligent and automated digital microfluidic control. Future studies may further enhance algorithm adaptability and hardware real-time deployment capabilities, while expanding into areas such as multi-droplet collaborative optimization and automated scheduling of biochemical experimental workflows.

## 6. Conclusions

This paper addressed the test path planning problem in digital microfluidic biochips and proposed an integrated optimization framework that combines a priority-based representation of test paths with an improved sparrow search algorithm. By mapping discrete droplet motion and fluidic constraints into a continuous priority space, the framework reformulates test path planning as a continuous optimization problem and enables the use of meta-heuristic search while preserving the physical feasibility of the generated paths.

Extensive simulations on standard 7 × 7–15 × 15 benchmark chips via both offline and online test models show that the proposed method achieves offline test path lengths equal to the known Euler path lower bounds on these regular arrays and yields shorter or comparable online paths with improved stability compared with representative swarm-based algorithms. In particular, ISSA maintains favorable average and worst-case behavior and reduced variance as chip size increases, indicating good scalability and robustness under complex, time-varying fluidic constraints.

Future work will focus on experimental validation on real DMFB hardware, incorporating more detailed models of stochastic actuation faults, time-varying defects and droplet deformation, and extending the framework to high-density multi-droplet scenarios with collaborative scheduling and fault-tolerant obstacle avoidance. These directions are expected to further advance intelligent and reliable testing in digital microfluidic systems.

## Figures and Tables

**Table 3:** Schedule of single biomedical assay.

Chip Size	7 × 7	9 × 9	11 × 11	13 × 13
Time slots for movement	7	8	10	13
Time slots for mixing	20	50	80	140
Time slots for detection	50	71	81	121
Total time slots	77	129	171	274

**Table 4:** Schedule of multiple biomedical assays for 15 × 15 array.

Time(s)	Time Slots	Operation
0	0	Sample2 and reagent 2 begin to move towards the mixer.
0.8	13	Sample 2 and reagent 2 start to mix together.
6.0	96	Sample 2 and reagent 2 continue mixing. Sample 1 and reagent 1 begin to move towards the mixer.
6.8	109	Sample 2 and reagent 2 end mixing and mixture 2 begin to move towards the optical detection site 2. Sample 1 and reagent 1 start to mix together.
12.8	205	Mixture 2 continues the optical detection. Sample 1 and reagent 1 end mixing and mixture 1 begin to move towards the optical detection site 1.
19.8	317	Mixture 2 ends optical detection and leaves to the sink. Mixture 1 continues the optical detection.
25.8	413	Mixture 1 ends optical detection and leaves to the sink. The biomedical assays are complete.

**Table 6:** Comparison of ISSA and IGWO algorithms in stability of DMFB online test path.

Algorithm	Chip Size	Minimum Value	Maximum Value	Mean Value	Standard Deviation
IGWO [25]	7 × 7	95	99	96.5	0.78
	9 × 9	159	162	159.5	0.79
	11 × 11	239	244	239.8	1.18
	13 × 13	335	344	339.8	1.83
	15 × 15	449	471	459.0	3.43
ISSA	7 × 7	94	94	94	0
	9 × 9	158	158	158	0
	11 × 11	238	239	238.08	0.27
	13 × 13	334	336	334.52	0.86
	15 × 15	446	455	450.78	2.08

## Data Availability

The original contributions presented in this study are included in the article. Further inquiries can be directed to the corresponding author.
